# Evaluation of a rapid colorimetric field test to assess the effective life of long-lasting insecticide-treated mosquito nets in the Lao PDR

**DOI:** 10.1186/1475-2875-12-57

**Published:** 2013-02-07

**Authors:** Michael D Green, Mayfong Mayxay, Ray Beach, Tiengkham Pongvongsa, Samlane Phompida, Bouasy Hongvanthong, Viengxay Vanisaveth, Paul N Newton, Lucrecia Vizcaino, Isabel Swamidoss

**Affiliations:** 1 Division of Parasitic Diseases and Malaria, Centers for Disease Control and Prevention, Atlanta, GA, USA; 2 Lao-Oxford-Mahosot Hospital-Wellcome Trust Research Unit, Microbiology Laboratory, Mahosot Hospital, Vientiane, Lao PDR, Laos; 3 Faculty of Postgraduate Studies, University of Health Sciences, Vientiane, Lao PDR, Laos; 4 Centre for Clinical Vaccinology and Tropical Medicine, Churchill Hospital, Nuffield Department of Medicine, University of Oxford, Oxford, UK; 5 Savannakhet Provincial Malaria Station, Savannakhet Province, Lao PDR, Laos; 6 Centre of Malariology, Parasitology and Entomology, Ministry of Health, Vientiane, Lao PDR, Laos

**Keywords:** Permanet, ITN, LLIN, Deltamethrin, Colorimetric, Bioassay, Mosquito, Malaria, Net, Lao PDR, Laos

## Abstract

**Background:**

Malaria morbidity and mortality have been significantly reduced through the proper use of insecticide-treated mosquito nets, but the extra protection afforded by the insecticide diminishes over time. The insecticide depletion rates vary according to location where wash frequency and wear are influenced by cultural habits as well as the availability of water. Monitoring of available insecticides on the net surface is essential for determining the effective life of the net. Therefore, a rapid and inexpensive colorimetric field test for cyanopyrethroids (Cyanopyrethroid Field Test or CFT) was used to measure surface levels of deltamethrin on insecticide-coated polyester nets (PowerNets™) in rural Lao PDR over a two-year period.

**Methods:**

Net surface levels of deltamethrin were measured by wiping the net with filter paper and measuring the adsorbed deltamethrin using the CFT. A relationship between surface levels of deltamethrin and whole net levels was established by comparing results of the CFT with whole levels assayed by high-performance liquid chromatography (HPLC). An effective deltamethrin surface concentration (EC_80_) was determined by comparing mosquito mortality (WHO Cone Test) with CFT and HPLC results. Five positions (roof to bottom) on each of 23 matched nets were assayed for deltamethrin surface levels at 6, 12, and 24 months. Mosquito mortality assays (WHO Cone Tests) were performed on a subset of eleven 24-month old nets and compared with the proportion of failed nets as predicted by the CFT.

**Results:**

At six months, the nets retained about 80% of the baseline (new net) levels of deltamethrin with no significant differences between net positions. At 12 months, ~15-40%, and at 24 months <10% of deltamethrin was retained on the nets, with significant differences appearing between positions. Results from the CFT show that 93% of the nets failed (deltamethrin surface levels </= EC_80_) at 24 months. This value is in agreement with 91% failure as determined by the WHO Cone Test on a subset of 11 nets. The CFT results show that 50% of the nets from Laos failed at 12 months of normal use.

**Conclusion:**

The CFT is a useful and accurate indicator of net efficacy and may be substituted for mosquito bioassays.

## Background

Routine and correct use of insecticide-treated mosquito nets (ITNs) reduces morbidity and mortality from malaria [1]. The insecticide provides extra protection against mosquito biting, especially for nets which have been compromised [2]. However, washing reduces the amount of insecticide until the ITN no longer provides additional protection [3]. If replacement is delayed beyond the effective life of the ITN, malaria risk increases. Since frequency of ITN washing varies depending, for example, on the availability of water and cultural habits, ITN programs need a practical way to monitor effective net life.

Improvements in insecticide application techniques have resulted in new ITNs, particularly the long-lasting insecticidal net (LLIN), which has very high wash resistance [4]. A brand of LLIN, in extensive use worldwide, is PermaNet 2.0™. This net consists of multifilament polyester coated with a formulation of deltamethrin. In a large-scale field assessment in Uganda, PermaNet™ 2.0 retained insecticide well (41.5% of baseline dose) and maintained high bioactivity after 3 years of use [5].

Comprehensive field evaluations of ITNs in developing countries are few, due to the lack of resources such as chromatographic instrumentation for chemical analysis and insectaries for mosquito bioassays.

A simple, inexpensive, non-destructive, and field-adaptable technique for measuring deltamethrin levels on the surface of deltamethrin-coated polyester nets has been developed [6]. This technique, the cyanopyrethroid field test (CFT), correlated well with mosquito bioassay results (WHO Cone Test) and is suggested to be a good indicator of net efficacy [6]. In the Lao PDR (Laos), use of ITNs and artemisinin combination therapy (ACT) has contributed to reduced malaria incidence and deaths. The number of people sleeping under ITNs increased from 25% in 1999 to 60% in 2002 and death rates from malaria fell from 9 per 100,000 in 1990 to 0.4 in 2006 [7]. A net evaluation study in Laos suggested that the use of ITNs was associated with reduced mosquito density relative to sites where untreated nets were used. Also, the parous rate of *Anopheles dirus* decreased to almost 0% in the village where ITNs were distributed, while the rate for the site with untreated nets did not change after net introduction [8]. Therefore, nets treated with insecticide are likely to provide protection against most mosquito bites but for ITNs to continue to be effective, insecticide levels should be monitored and maintained. The objective of this study was to investigate the loss of insecticide from deltamethrin-treated polyester bed nets distributed in rural Laos and to predict time to failure of efficacy by using the CFT technique.

## Methods

### Evaluation of nets used in Laos

**Figure 1:**
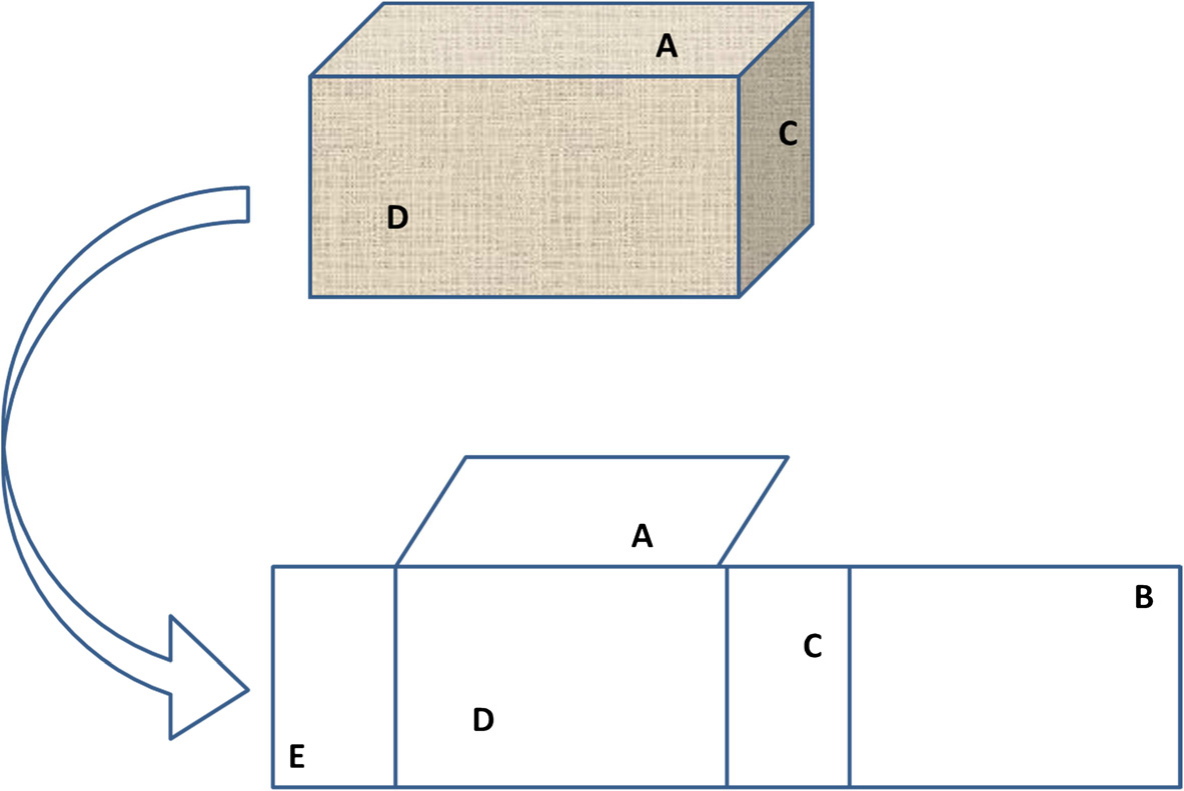
**Sampling locations used for a rectangular type bed net. **“**A**” is the roof and “**E**” is the lower bottom edge.

PermaNets 2.0™ (Vestergard-Frandsen, rebranded as PowerNets™) were distributed to households in rural Xieng Lai Khok village (16.673° N 105.652° E; 180 m above msl), Phalanxay District, Savannakhet Province, southern Laos in 2007. From April 2007 to October 2009, the mean ambient temperature in a house was 25.7 (95% CI, 25.6-25.8)°C. Fifty nets were tagged with identifying numbers. At 6, 12, and 24 months, the nets were brought to a central location in the village for deltamethrin testing using sampling and colorimetric techniques (CFT) [6]. Five positions (A, B, C, D, and E) on the net were assayed (Figure 1) at 6, 12, and 24 months, respectively. There were 23 matched nets with data for all three time points. At six and 24 months, both sampling and colorimetric testing were completed on-site in Laos. At 12 months, the sample collection was performed in the field, while the colorimetric testing was performed at CDC. It was assumed that all nets distributed at time zero (baseline) were within deltamethrin content specifications (55 mg/m^2^) for a new, unused net. Eleven randomly-selected nets from the 24-month collection underwent bioassay using the WHO Cone Test. Half-lives were determined from an exponential decay curve (y = ae^-x^). In 2007 and 2008, the head each household possessing a net was questioned as to how many times the net was washed/year.

### Net assay by the cyanopyrethroid field test (CFT)

The cyanopyrethroid field test (CFT) was performed as described by Green et al. [6]. Two 13-mm diameter Whatman 597 filter paper disks were wiped with consistent pressure using magnets (magnetizing force = 35000 Oersted) 30 times across 90 mm of net material; one on the interior and one on the exterior surface. The use of magnets maintains a consistent pressure of filter papers against the surface of the net material. The combined amount of deltamethrin adsorbed onto both filter paper disks (both sides of net) was measured by comparison with filter paper disks containing known quantities of deltamethrin (calibration standard). Pairs of sample disks as well as calibration disks containing known amounts of deltamethrin were placed in 24-well polystyrene flat-bottomed tissue culture plates. A 0.2 ml aliquot of a solution containing 30 mg/ml of 1,2-dinitrobenzene and 4-nitrobenzaldehyde dissolved in methyl cellosolve (2-methoxyethanol) was added to each well. After allowing the disks to soak in the reagent for five minutes, the colorimetric reaction was activated with the addition of 0.05 ml 0.4 N sodium hydroxide. The reaction was allowed to proceed for five minutes whereupon the intensity of the purple color was recorded by digital photography using a standard digital camera. Deltamethrin concentrations were determined by comparing the color intensity of the sample disks to the calibration standards using image analysis techniques as described by [6].

### Correlation of surface levels (CFT) with entire net levels (HPLC)

Total net deltamethrin levels were determined from complete extraction of deltamethrin from the net material followed by analysis using a modified high-performance liquid chromatographic (HPLC) technique (CIPAC/4838). The relationship between deltamethrin surface levels and deltamethrin total net levels was established using the following method. Insecticide-diminished nets were prepared by soaking 30 × 30 cm sections for five minutes in varying concentrations of aqueous 2-propanol. The sections were subsequently blotted and air-dried. Briefly, 5 × 5 cm portionswere cut from each quadrant, weighed and transferred to glass vials containing 15 ml of extraction solvent (20% 1,4-dioxane, 80% iso-octane). Samples were sonicated for 15 minutes followed by rotation (40 rpm). The predominant “S” isomer of deltamethrin was effectively separated from the inactive “R” isomer using a 4.6 × 150 mm, 5micron Silica column with the mobile phase consisting of 98% iso-octane, 2% 1,4-dioxane with a flow rate of 1.5 ml/min and column temperature of 30°C. Detection was accomplished using UV absorption at 230 nm. Both S and R isomers of deltamethrin were combined and calculated per weight of net material, then adjusted to represent mass per area (mg/m^2^) by using the conversion factor of 30 g/m^2^ for a 75 denier net. CFT values (μg/sample) and whole net deltamethrin levels (mg/m^2^) were plotted and a CFT value equivalent to an unused net containing the standard manufacturer’s declared concentration of 55 mg/m^2^ determined from linear regression analysis. CFT values are subsequently described as the percentage of surface deltamethrin concentration relative to an unused net.

### Bioassay (WHO cone test)

Four standard WHO mosquito bioassay cones were fastened to a 30 × 30 cm section of net and ~ five susceptible, non-blood fed, 2–5 day old *Anopheles gambiae* s.s. KISUMU1 strain females were aspirated into each cone (WHO 2005). After exposure to the net for three minutes, the mosquitoes were transferred to a clean pint-sized mosquito cage and held for 24 hours with access to sugar solution. The cone tests were performed in duplicate, resulting in the exposure of a total of at least 40 mosquitoes for each LLIN section tested (10 females per quadrant). The % mortality for each net section was determined from the proportion of dead mosquitoes relative to the total number exposed. Untreated nets were used as controls. Bioassays were performed on the deltamethrin-diminished nets and 24-month old Lao nets.

### Determination of EC_80_

WHO guidelines for the Cone Test suggest an effective net should have surface insecticidal levels strong enough to kill at least 80% of a susceptible vector [9]. The effective concentration (EC_80_) where deltamethrin surface levels approximate 80% mosquito mortality (WHO Cone Test) was determined by plotting % mortality as a function of total net deltamethrin levels from a combination of bioassays performed in our lab and data from published studies using similar type nets and mosquitoes. Nine nets of varying whole net deltamethrin concentrations (confirmed by HPLC analysis) were assayed in our lab using the CFT and WHO cone test as described above. Published reports of whole net levels (mg/m^2^) associated with the WHO Cone Test bioassays were converted to CFT values (μg/sample) using the relationship y = 0.0472× – 0.1239 determined by the comparison of whole net levels (mg/m^2^) with CFT values (μg/sample) as described above. A four-parameter logistics curve (Y = Y_0_ + a/(1 + e^-(X-Xo/b)^) was fitted to the data using Sigmaplot® software (Systat Software, Inc., San Jose, CA USA) from which the EC_80_ was determined.

## Results and discussion

**Figure 2:**
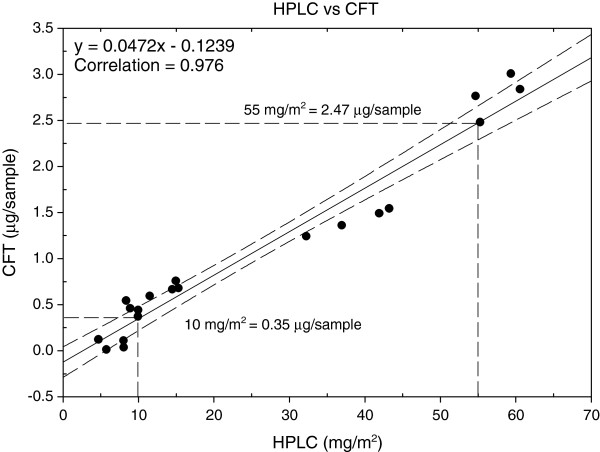
**Relationship between CFT deltamethrin values and whole net deltamethrin values as determined by HPLC. **(------------ 95% C.**I**.).

**Figure 3:**
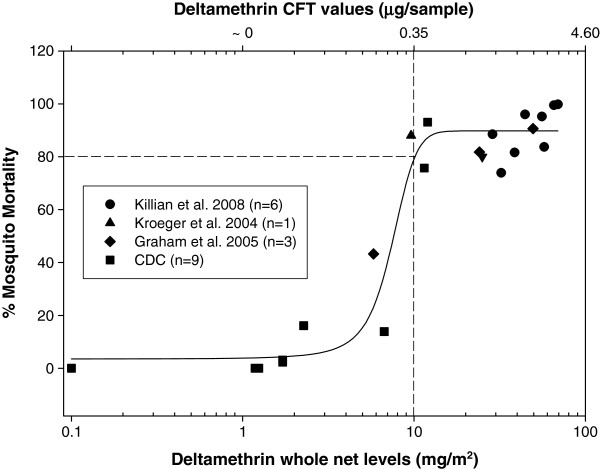
**Four**-**parameter logistics curve used to determine the effective whole net deltamethrin levels representing 80% mosquito mortality (EC**_**80**_**).**

**Figure 4:**
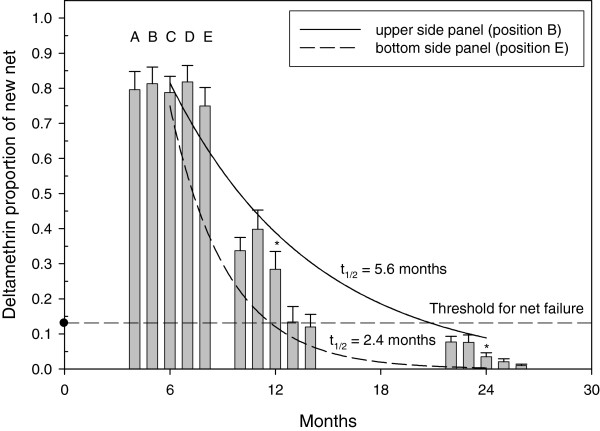
**Plot of mean deltamethrin surface levels for each location sampled on a net as a function of time (n = 23 matched nets).** * Deltamethrin surface concentrations were significantly different between net positions at 12 and 24 months (p < 0.001 ANOVA). Threshold proportion for net failure (EC_80_) is 0.14.

**Figure 5:**
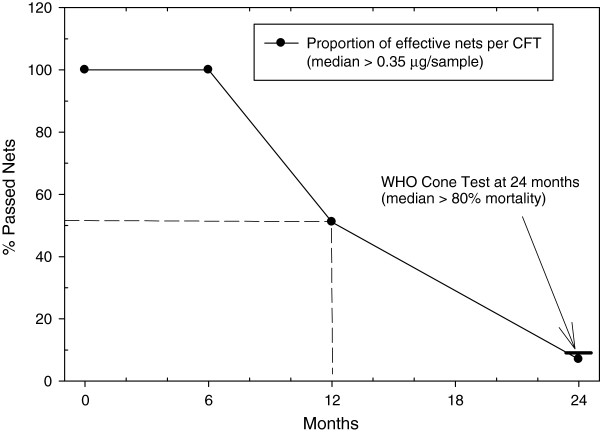
**Prediction of net failure using CFT values. **Net failure via Cone Test is defined as the median of all 5 positions </= 80% mosquito mortality. The % failure via the CFT (n = 43, 93%) was not significantly different than that obtained via the WHO Cone Test (n = 11, 91%).

Figure 2 shows the linear relationship between CFT deltamethrin levels (μg/sample) and whole net deltamethrin levels (mg/m^2^) as determined by HPLC. A Bland-Altman plot [10] revealed the mean ratio between the HPLC and CFT methods to be 0.03816 (95% CI, 0.03051-0.04582). Therefore, on average, about 3.8% of the total content of deltamethrin for this type of net (PermaNet2.0™) is on the surface. This ratio may be different on other types of net. The surface deltamethrin levels, as determined by the CFT, corresponding to the total amount of deltamethrin typically present in a new unused net (55 mg/m^2^) is 2.47 μg/sample (95%CI, 2.29-2.67). A whole net deltamethrin EC_80_ value of 10 mg/m^2^ was determined from a logistics plot (Figure 3). Using the linear relationship derived in Figure 2, the surface deltamethrin level (CFT) equivalent to 10 mg/m^2^ is 0.35 μg/sample. Therefore, a net containing less than 14% (0.35/2.47 = 0.14) deltamethrin relative to a new net will be designated as the threshold value representing a failed net. The deltamethrin surface levels derived from the CFT are represented as a proportion relative to a new (baseline) net. Figure 4 shows the average deltamethrin proportion for within-net location (A, B, C, D, E) at the three time points for 23 matched nets. After 6 months, ~80% of surface deltamethrin levels remained on the net with no significant differences between net locations. At 12 and 24 months, significant differences in deltamethrin proportion (p < 0.001, ANOVA) between net positions are apparent. This is presumably a result of net surface contact with bedding material and people, with the lower portion of the net (position E) having the most exposure. The estimated deltamethrin half-life for the bottom position (E) was 2.4 months and 5.6 months for position B (top side panel). It was observed that many households stored blankets on top of the net (position A), probably explaining the lower proportion in position A relative to position B at 12 months. A comparison of the relationship between individual position CFT values and the median CFT values for all five positions per net revealed position C (middle side panels) as having the best correlation (R = 0.95). Therefore, it is suggested that sampling of position C may be sufficient to represent the deltamethrin concentration for the entire net. Figure 5 shows the progression of % failed nets over time as a function of deltamethrin surface levels. Failed nets were defined as: 1) CFT median of all 5 positions </= EC_80_, and 2) CFT value at position C </= EC_80_. Eleven 24-month field-exposed randomly chosen nets used in Laos were subjected to the WHO cone test and 90.9% failed as determined by median mosquito mortality. The percent failure predicted by the CFT of 43 nets was 93% (median), and 86% (position C). The CFT predictions were closely in agreement with 91% (n = 11) net failure as determined by the Cone test bioassay. In comparison, Permanet™ 2.0 used for 2 years in Ethiopia showed 67-72% mortality against *Anopheles arabiensis*[11] and Permanet 2.0 nets in Uganda showed 74% mosquito mortality after two years of use [5]. Therefore, nets from these studies would also be considered as failed (% mortality </= 80) at two years of field use. The CFT shows that ~20% and ~50% of the nets used in Laos failed at about 9 and 12 months, respectively. For all 50 nets, median (range) washing frequency was 1 (1–2) for 2007 and 1 (1–5) for 2008. The washing frequency (n = 50) was slightly higher in 2007 and 2008 with a median of 1.5 (range 1–2) and 2.0 (range 1–5), respectively.

## Conclusions

The CFT was shown to be useful as an indicator of net efficacy in a developing country where resources for chemical and biological analyses are lacking. This sampling process, in which residual insecticide from the surface of the net is measured, may be a more accurate indicator of insecticide bioavailability than measuring levels within the fabric. One advantage of the CFT is the ability to screen larger numbers of nets rapidly and inexpensively, although it is recommended that the WHO Cone Test should be used to confirm a failed net when possible. The higher deltamethrin levels at the top of the net suggest that contact of the net material with bedding or other materials have a significant contribution to insecticide depletion rates. Currently the CFT is applicable to cyanopyrethroid-coated polyester nets, such as the PermaNet™. Due to variability in frequency of washing as well as technique, it is suggested that statistically relevant subsets of distributed nets be monitored quarterly to assess trends in net efficacy. These trends can be useful in predicting when ineffective nets are to be replaced.

## Competing interests

The authors declare that they have no competing interests.

## Authors’ contributions

MDG conceived the study, performed the colorimetric testing and data analysis and drafted the manuscript. MM coordinated the study, assisted with sampling and assisted with drafting the manuscript. RB assisted with drafting the manuscript. TP, SP, BH and VV assisted with sampling and colorimetric testing. PN assisted with the coordination of the study and drafting the manuscript. LV and IS performed the mosquito bioassays. All authors read and approved the final manuscript.
